# Supplementary material for: Visualization of Bacillus subtilis spore structure and germination using quick-freeze deep-etch electron microscopy
Source: Microscopy (Oxf). 2024 Apr 25;73(6):463–72. doi: 10.1093/jmicro/dfae023 (PMC11630275; doi:10.1093/jmicro/dfae023)
Supplement: dfae023_Supp [file dfae023_supp.zip › suppl_data/Certificate_of_editing-BNXKQ_32_qa_aurcpkx.pdf]

Editing Certificate

This document certifies that the manuscript listed below has been edited to ensure language and grammar accuracy and is error free in these aspects. The edit was performed by professional editors at Editage, a brand of Cactus Communications. The author's core research ideas were not altered in any way during the editing process. The quality of the edit has been guaranteed, with the assumption that our suggested changes have been accepted and the text has not been further altered without the knowledge of our editors.

MANUSCRIPT TITLE

Visualization of Bacillus subtilis Spore Structure and Germination by Quick-Freeze-Deep Etch Electron Microscopy

AUTHORS

Kiran Jalil, Yuhei O. Tahara, Makoto Miyata

ISSUED ON

February 27, 2024

JOB CODE

BNXKQ_32

THE EDITAGE Promise QUALITY GUARANTEE

Prabh Grewal
Senior Vice President - Editage

editage | helping you get published

Since 2002, Editage has helped over 430,000 authors publish around 1.2 million research papers in scholarly journals across over 1000 disciplines through editorial, translation, transcription, and publication support services. Editage is a brand of Cactus Communications (cactusglobal.com), a science communication and technology company.

CACTUS

GLOBAL :
+1(833) 979-0061 | request@editage.com

JAPAN :
0120-50-2987 | submissions@editage.com
